# Switching to the aflibercept (3 mg) therapy for treatment-resistant wet age-related macular degeneration: 1-year outcomes

**DOI:** 10.1097/MD.0000000000037839

**Published:** 2024-04-19

**Authors:** Chengcheng Feng, Wenjuan Chu, Ping Lin, Haifeng Xu, Xiuli Chen

**Affiliations:** a Qingdao Eye Hospital, Shandong First Medical University, Qingdao, China; b State Key Laboratory Cultivation Base, Shandong Provincial Key Laboratory of Ophthalmology, Shandong Eye Institute, Shandong First Medical University, Qingdao, China.

**Keywords:** age, anti, dose aflibercept, high, related macular degeneration, related macular degeneration, VEGF, wet age

## Abstract

This study aimed to elucidate 1-year outcomes following switching to the aflibercept (3 mg) therapy for treatment-resistant wet age-related macular degeneration (wAMD). In this prospective, open-label, non-controlled clinical trial, 18 patients with wAMD who had multiple recurrences or persistent exudation despite intravitreal injections of anti-vascular endothelial growth factor agents (except aflibercept) received a 3-mg intravitreal aflibercept injection every 4 weeks. Each patient received 3 to 8 injections. The central retinal thickness and fibrovascular pigment epithelial detachment height decreased significantly at 1 month after initiation of the aflibercept injection, and the values were 146 and 163.2 μm, respectively, at the final visit. The morphological improvement was sustained. The intraretinal and subretinal fluid was completely absorbed at the end of the follow-up. The logMAR vision increased from baseline 0.68 to 0.59 (*P* < .05). No ocular or systemic adverse events occurred. The intravitreal injection of 3-mg aflibercept seems to be feasible in the treatment of wAMD unresponsive to other anti-vascular endothelial growth factor agents.

## 1. Introduction

Age-related macular degeneration (AMD) is a leading cause of irreversible visual impairment in elderly individuals and ranks as the third most common cause of blindness globally. Wet AMD (wAMD), also known as advanced neovascular AMD, usually leads to faster and more severe vision loss. Currently, the first-line treatment for wAMD is the intravitreal injection of anti-vascular endothelial growth factor (VEGF) drugs. However, patients may have no response or poor response to repeated anti-VEGF injections.^[1]^ To better reduce the progression of macular degeneration, many treatment strategies have been adopted, such as administration of different anti-VEGF drugs through different routes, increasing the therapeutic dose of anti-VEGF agents, and using an add-on therapy.^[2–4]^

Aflibercept is a soluble decoy receptor fusion protein, consisting of part of the VEGF receptors’ structure, VEGFR-1 and VEGFR-2, which can bind to all isoforms of VEGF-A, as well as the placental growth factor, blocking its downstream function. The binding affinity of intravitreal aflibercept to VEGF-A was reported to be greater than that of bevacizumab or ranibizumab.^[5]^ The greater affinity to the VEGF-A could translate into a higher efficacy in the treatment of wAMD. As predicted by a mathematical model, the drug action might be sustained for a longer duration in the eye,^[6]^ not requiring frequent dosing. A clinical trial also supported these findings.^[7]^ The molar concentration of aflibercept was 348 nmol/L, which was 4.97 times higher than that of ranibizumab (70 nmol/L) and 1.67 times higher than that of conbercept (208 nmol/L). Three intravitreal injections of aflibercept at a dosage of 2 mg each time markedly reduced the central retinal thickness (CRT) and the volume of retinal fluid as observed using optical coherence tomography (OCT) in patients with exudative neovascular AMD, but no clinically or statistically significant changes in the visual acuity (VA) were noted, which may be related to the persistent macular edema. This study aimed to prospectively evaluate the 1-year VA and other anatomical parameters of intravitreal higher-dose (3 mg) aflibercept injections in patients who suffered wAMD which was resistant to other anti-VEGF agents.

## 2. Methods

This prospective study was conducted according to the guidelines of the Declaration of Helsinki and approved by the Ethics Committee of Qingdao Eye Hospital of Shandong First Medical University (2021-50). Informed consent was obtained from all subjects involved in the study.

### 2.1. Study population

Eighteen patients (18 eyes) who had no response or poor response to previous treatments with a minimum of 3 intravitreal injections (once a month in the last 3 months) of anti-VEGF agents other than aflibercept for wAMD were recruited between December 2021 and March 2022. Three eyes had type 1 neovascular AMD, while 15 eyes had polypoidal choroidal vasculopathy, which is also called aneurysmal type 1 neovascular AMD. They all had persistent intraretinal fluid (IRF) or subretinal fluid (SRF), or both, on spectral-domain OCT images.^[8,9]^ The exclusion criteria were as follows:^[10,11]^ uncontrolled intraocular pressure (IOP) of more than 25 mm Hg; existing vitreous hemorrhage or inflammation; anti-VEGF therapy within 30 days before the initiation of this study; prior photodynamic therapy treatments; previous intraocular surgeries; and other retinal conditions, including macular hole, vitreomacular traction, epiretinal membrane, retinal detachment, pseudo vitelliform macular dystrophy, and peripapillary choroidal neovascularization (CNV).

### 2.2. Intravitreal injections of aflibercept

Intravitreal administration of aflibercept was performed in a sterile laminar flow operating room following sterile surgical procedures. A 30-gauge needle was inserted from the superotemporal quadrant, 3.5 to 4.0 mm from the corneoscleral limbus, to inject 3-mg/0.075-mL aflibercept into the vitreous cavity. The injection was administered once every 4 weeks until the retina became dry (no evidence of IRF or SRF on OCT images). Since all eyes were not treatment-naive and had received an average of 4.9 (range, 3–10) ranibizumab or conbercept injections and a minimum of 3 injections within 3 months prior to switching to aflibercept, 3 to 5 loading doses of aflibercept were not considered.

When there was at least one of the following conditions, patients continued receiving aflibercept injections on an individualized basis as a modified or extended treatment regimen: recurrent or persistent IRF or SRF on OCT images, the appearance of new hemorrhage on color fundus photographic or ophthalmoscopic images, or decreased visual acuity as compared to the previous examination. Retinal pigment epithelial detachment (PED) was not an indication for further treatment.

### 2.3. Baseline and follow-up examinations

Before (baseline) and at 1, 3, 6, and 12 months after initiating the aflibercept treatment, the VA was measured using the Snellen chart and converted into the logarithm of minimum angle resolution (logMAR). The IOP was measured with a Goldmann applanation tonometer. Slit lamp biomicroscopy and funduscopy were also performed. Severe post-injection complications, including intraocular inflammation, infection, and systemic arteriothrombotic events, like stroke and myocardial infarction, were recorded.

Fundus fluorescein angiography and indocyanine green angiography were performed using the Heidelberg Retina Angiograph 2 (Heidelberg, Germany). OCT (Optovue RTVue XR, Optovue, Fremont, CA) was used to radially scan the macular area with an angular interval of 20°. On the spectral-domain OCT images, the CRT was measured as the distance between the internal limiting membrane and the Bruch membrane in the central fovea. The maximum pigment epithelial detachment height (PEDH) was measured as the distance between the outer border of the retinal pigment epithelium (RPE) and the inner border of the Bruch membrane in the area of CNV. These measurements were triplicated by the same experienced physician, and the average value was calculated. A retinal specialist who was masked to clinical information, including visual acuity, treatment history, and treatment dosage, evaluated all images. If the cystoid dark area of the retina between the layers of the macular area disappeared, it was judged that the IRF was completely absorbed. The complete disappearance of the subretinal dark area in the macular region was considered as complete absorption of the SRF.

### 2.4. Statistical analysis

SPSS version 22.0 (IBM, Armonk, NY) software was used for statistical analysis. The continuous variables were expressed as mean ± standard deviation (x̄ ± s), and the changes before and after treatment were expressed as mean difference and 95% confidence interval (CI). The BCVA, CMT, and PEDH before and after treatment were compared using the paired *t* tests. A *P* value of < .05 was considered statistically significant.

## 3. Results

### 3.1. Patient demographics

The mean age of all patients was 69.33 ± 5.99 years. Fourteen patients were men, and 4 patients were women. The number of intravitreal injections of aflibercept was 3 to 8 for each affected eye. At 3, 6, and 12 months after switching to the aflibercept treatment, the average number of injections was 2.5, 3.7, and 5.1 in all eyes, respectively.

**Figure 1.**
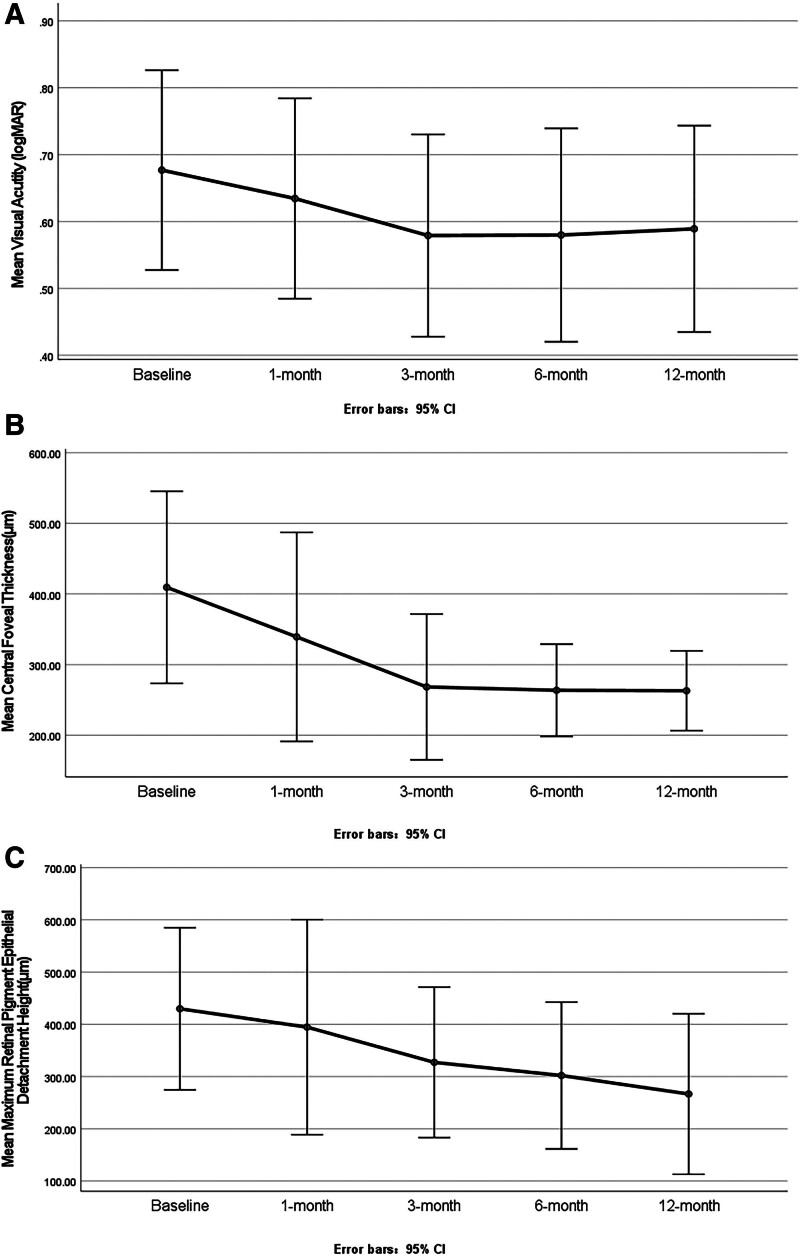
Mean visual acuity (A), central foveal thickness (B), and maximum retinal pigment epithelial detachment height (C) before and after initiating the 3-mg aflibercept treatment. CI = confidence interval, logMAR = logarithm of the minimum angle resolution.

Compared to the average visual acuity (logMAR) of 0.68 before starting the aflibercept injections, 8 eyes (44.4%) had BCVA improved for more than or equal to one line, 8 eyes (44.4%) had an unaltered vision, and 2 eyes (11.1%) presented a line of visual loss at 1 month after the add-on treatment. The average vision was 0.63 ± 0.29, 0.58 ± 0.29, 0.58 ± 0.31, and 0.59 ± 0.30 at 1, 3, 6, and 12 months, respectively (Fig. 1A). The average IOP was 14.8 ± 3.6 (8–21) mm Hg at the baseline versus 16.7 ± 2.8 (10–21) mm Hg at the final visit. No obvious IOP increase occurred during the follow-up period.

### 3.2. Evaluation of the CRT and PEDH

**Figure 2.**
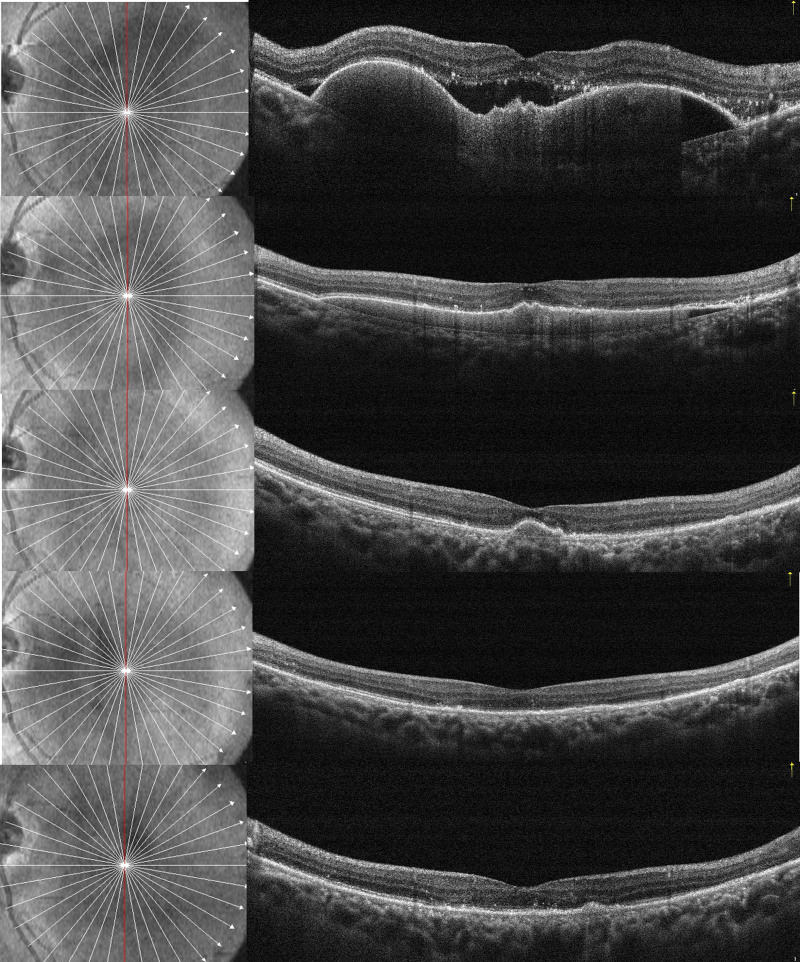
A case of a 69-year-old man with persistent exudation who had received 5 conbercept injections before switching to the aflibercept (3 mg) therapy. Optical coherence tomographic scans going through the fovea at the baseline, 1, 3, 6, and 12 months after switching showed a decrease in the height of the subfoveal fibrovascular pigment epithelial detachment and a gradual resolution of the subretinal fluid.

The mean CRT decreased significantly from 409 ± 55.55 μm at the baseline to 339 ± 60.45 μm at 1 month (*P* < .05), 268 ± 42.15 μm at 3 months (*P* < .05), 263 ± 26.71 μm at 6 months (*P* < .05), and 263 ± 23.08 μm at 12 months after initiating the aflibercept injections (*P* < .05) (Fig. 1B). All eyes suffered fibrovascular PED, and 2 eyes also had hemorrhagic PED. The height of fibrovascular PED decreased from 429.9 ± 63.42 μm at the baseline to 394.7 ± 84.14 μm (*P* < .05) at 1 month, 327.29 ± 58.87 μm at 3 months (*P* < .05), 302.14 ± 57.39 μm at 6 months (*P* < .05), and 266.71 ± 62.81 μm at 12 months (*P* < .05) (Fig. 1C). The CRT decreased to the lowest level at 6 months and maintained almost unchanged thereafter, whereas the PEDH decreased to the lowest level at 12 months (Fig. 2).

### 3.3. Evaluation of the IRF and SRF

Eight eyes had the IRF, while 18 eyes had the SRF at the baseline. At 1, 3, 6, and 12 months after the treatment, the complete absorption of IRF was seen in 4 eyes (50%), 4 eyes (50%), 5 eyes (62.5%), and 8 eyes (100%), respectively; the complete absorption of SRF was observed in 6 eyes (33.33%), 14 eyes (77.78%), 16 eyes (88.89%), and 18 eyes (100%), respectively (Fig. 2). Both the IRF and SRF significantly decreased at 1 and 12 months. The proportion of eyes that became completely dry at each follow-up timepoint was 33.33%, 77.78%, 88.89%, and 100%, respectively. At the last visit, 100% of eyes were completely dry, and the mean CRT was 263 ± 23.08 μm, which was significantly thinner than the baseline value (*P* < .05).

### 3.4. Injection frequency

Ten patients (55.6%) had 2 injections, and 8 patients (44.4%) received 3 injections with an interval of 1 month, after which the interval to the subsequent injection was 8 to 12 weeks in 15 patients and 6 months in 2 patients. One patient with the RPE tear was injected 5 times consecutively. In average, there were 3.7 injections within 6 months and 5.1 injections within 12 months.

### 3.5. Complications

During the follow-up period, 2 eyes developed subconjunctival hemorrhage. No local or systemic complications, such as persistent elevated IOP, endophthalmitis, and cardiovascular and cerebrovascular circulatory disturbance, occurred in all affected eyes. The RPE tear was observed in 1 eye at 1 month after the aflibercept treatment.

## 4. Discussion

It remains unclear why some patients with wAMD respond well to anti-VEGF treatment, but some do not. Based on the mathematical modeling, even though the initial response to anti-VEGF treatment seems good, over time the disorder can become refractory or recurrent, which may be due to the drug resistance or pharmacokinetic tolerance to anti-VEGF agents,^[11,12]^ or the decrease in drug sensitivity because of the changes in neovascular structures. The drug tolerance might be associated with the increase of VEGF expression, the increase of VEGF receptor expression, the modification of signal transduction, or the change of CNV growth-stimulating factors. Switching to the aflibercept treatment was reported to be effective in patients with bevacizumab or ranibizumab resistance.^[13–15]^ Nevertheless, 45% of the affected eyes could not maintain the retinal dryness with a conventional dose of aflibercept (2 mg) once every 2 months. When the treatment protocol was changed to the same dose once a month, the therapeutic effect was still not ideal.^[16]^ A recent phase 2 clinical trial disclosed the safety and efficacy of 8-mg aflibercept for treatment-naive patients with neovascular AMD,^[17]^ but refractory AMD has not been involved.

In this study, we increased the 2-mg commercially available aflibercept dose to 3 mg, achieving favorable effectiveness in the treatment of refractory wAMD that did not respond to conventional anti-VEGF treatment or had persistent exudation. The CRT, PEDH, IRF, and SRF were significantly decreased 1 month after the aflibercept treatment. At 1 year, 66% of the treated eyes improved their vision by 1 or more lines, and 33% of the eyes improved their vision by 3 or more lines. This indicates that not only the anatomical structure but also the visual function may be greatly improved in patients after receiving the aflibercept add-on treatment. During the follow-up period, there were no adverse reactions in the eye and the whole body.

Pharmacokinetic tolerance can occur due to the decrease in the number of drug molecules reaching the affected site.^[9]^ Most of the patients with refractory wAMD also suffer polypoidal choroidal vasculopathy, with a large area of PED. The drugs need to penetrate the RPE layer to reach the neovascular lesions. In patients whose lesions are not alleviated after the treatment with a standard dose, the rising dose may ensure adequate medication for the lesions.^[18]^ In addition, with the increasing treatment times, the level of VEGFs in the eyes might keep high, so a higher dose is possibly needed to reach the effective drug concentration.^[19]^ Chan et al^[20]^ investigated the prognosis of wAMD with neovascular PED treated with different doses of ranibizumab. Although the 2-mg group achieved rapid improvements in the vision and anatomical structure within the first year of follow-up, there was no difference from the 0.5-mg group. However, the risk of RPE tear in the high-dose group was significantly increased. According to You et al,^[4]^ in patients with wAMD that did not respond well to an injection of 2-mg aflibercept every 4 weeks, the treatment protocol of injecting 4 mg of aflibercept every 4 weeks significantly reduced the CRT, PEDH, IRF, and SRF, and improved the vision or maintained visual stability without eye and systemic complications. Considering the possibility that the repeated higher-dose anti-VEGF treatments may accelerate macular geographic atrophy,^[21–23]^ the treatment with 3 mg of aflibercept was used in our series. The RPE tear was found in only 1 of 18 patients, with an incidence far lower than 10% to 20% as reported previously.^[24,25]^ All patients did not have local or systemic complications. The aflibercept add-on protocol adopted in this study seemed to be safe and reliable.

Moreover, the injection times of 3-mg aflibercept in our study were greatly induced as compared to the report by Ertan et al^[26]^ who used a 3 + PRN regimen with 9.09 ± 3.9 injections of aflibercept within 30 months for patients with wAMD not responding to 16.8 ± 8.8 injections of ranibizumab during 30.18 ± 16.78 months. In our series, the average number of injections was 3.7 in 6 months and 5.1 in 12 months.

## 5. Conclusions

Switching to the 3-mg aflibercept therapy for patients with wAMD who had poor responses to other anti-VEGF drugs could achieve favorable therapeutic effects. The average BCVA, CRT, and PEDH of the affected eyes were improved at each follow-up timepoint compared with the baseline values. With the more personalized treatment-extension protocol, aflibercept significantly improved the imaging indexes of patients and stabilized their vision. Further investigations may be needed with a larger sample size and a longer observation time for the promotion of this potential treatment option in clinical practice.

## Acknowledgements

The authors would like to thank all of patients for their contribution to this research.

## Author contributions

**Conceptualization:** Xiuli Chen, Haifeng Xu.

**Data curation:** Chengcheng Feng, Wenjuan Chu, Ping Lin.

**Formal analysis:** Xiuli Chen, Haifeng Xu, Chengcheng Feng, Wenjuan Chu.

**Funding acquisition:** Xiuli Chen.

**Writing – original draft:** Xiuli Chen, Chengcheng Feng.

**Writing – review & editing:** Ping Lin, Xiuli Chen.
